# Fibromyalgia is associated with increased odds of prior pain-precipitated relapse among non-treatment-seeking individuals with opioid use disorder

**DOI:** 10.1080/07853890.2024.2422050

**Published:** 2024-11-05

**Authors:** O. Trent Hall, Pooja Lagisetty, Johnathan Rausch, Parker Entrup, Megan Deaner, Steven E. Harte, David A. Williams, Afton L. Hassett, Daniel J. Clauw

**Affiliations:** a Department of Psychiatry and Behavioral Health, Ohio State University Wexner Medical Center, Columbus, OH, USA; b Department of Internal Medicine, Division of General Medicine, University of Michigan, Ann Arbor, MI, USA; c Center for Clinical Management and Research, Ann Arbor, VA, USA; d Department of Anesthesiology, Chronic Pain and Fatigue Research Center, University of Michigan Medical School, Ann Arbor, MI, USA; e Department of Internal Medicine, Division of Rheumatology, University of Michigan Medical School, Ann Arbor, MI, USA

**Keywords:** Central nervous system sensitization, opioid-related disorders, fibromyalgia, chronic pain

## Abstract

**Background/Objectives:**

Chronic pain is an opioid use disorder (OUD) treatment barrier and associated with poor outcomes in OUD treatment including relapse. Fibromyalgia is a chronic pain condition related to central nervous system substrates that overlap with the brain disease model of OUD. We know of no studies that have looked at non-treatment seeking individuals, to see if fibromyalgia might represent a barrier to OUD treatment. Given many non-treatment-seeking individuals previously attempted recovery before experiencing relapse, and chronic pain is a known precipitant of relapse, fibromyalgia might be a currently unappreciated modifiable factor in OUD relapse and, potentially, a barrier to treatment reengagement among those not currently seeking treatment. This study aimed to determine if fibromyalgia is associated with greater odds of agreeing that *‘I have tried to stop using opioids before, but pain caused me to relapse’ among non-treatment seeking individuals with OUD.*

**Methods:**

This cross-sectional study recruited non-treatment-seeking individuals with OUD (*n* = 141) from a syringe service program. Ordinal logistic regression was used to determine if the presence of fibromyalgia increased the odds of agreement with prior pain-precipitated relapse.

**Results:**

Fibromyalgia was identified in 35% of study participants and associated with 125% greater odds of strongly agreeing that pain had previously caused them to relapse, even after accounting for relevant covariates, including age, sex, depression, anxiety, OUD severity, and pain severity.

**Conclusions:**

This study provides early evidence that the presence of fibromyalgia may be associated with increased odds of pain-precipitated OUD relapse.

## KEY MESSAGE

- Fibromyalgia may be uniquely related to pain‑precipitated relapse of OUD.

## 1. Introduction

In the United States (US), 82% of people living with opioid use disorder (OUD) went without medication for OUD (MOUD) in 2022 [1]. During this same year, 79,770 Americans died of opioid overdose [2]. Given the exceedingly high mortality burden associated with opioid overdose in the US, barriers to OUD treatment among non-treatment-seeking populations are of pressing concern [3–7]. Many non-treatment-seeking individuals report prior OUD treatment engagement followed by relapse to opioid use [8, 9]. Therefore, prior failed treatment episodes and factors associated with relapse provide important context for understanding the lack of treatment engagement among non-treatment-seeking individuals with OUD.

Chronic pain is a known precipitant of OUD relapse and may be a significant barrier to OUD treatment engagement among non-treatment-seeking individuals with OUD (defined as not currently seeking or engaged in OUD treatment). As many as 62% of OUD patients report chronic pain, and OUD patients with chronic pain are more likely to relapse and experience overdose than those without chronic pain [10–13]. Among non-treatment-seeking individuals, the fear that pain will become unbearable after stopping opioids is associated with OUD treatment delay [14]. Despite these clinically important observations, poorly operationalized definitions of pain, measurement issues, and difficulty contacting non-treatment-seeking samples have limited the study of pain in OUD [15]. Emerging literature suggests that defining pain by clinical pain phenotype using validated measures might provide new insight into the complex relationship between chronic pain and OUD [16–18].

Until recently, studies of comorbid chronic pain and addictive disorders had not benefited from recent advancements in the mechanistic taxonomy of pain. In 2016, the International Association for the Study of Pain (IASP) introduced a new third pain phenotype, called ‘nociplastic pain’, in addition to existing phenotypes ‘nociceptive pain’ and ‘neuropathic pain’ [19]. Nociplastic pain is a pain phenotype that *‘arises from altered nociception’* related to dysfunctional nociceptive processing at multiple levels of the nervous system [20]. Intriguingly, the central nervous system (CNS) substrates of nociplastic pain overlap extensively with those of the brain disease model of addiction [16–19, 21, 22].

Fibromyalgia is the most well-studied nociplastic pain condition, and much of what is known about the nociplastic pain phenotype has been established from studies of fibromyalgia [19]. Our group recently published a series of studies employing a validated surrogate measure of nociplastic pain and fibromyalgia in the context of alcohol and substance use disorders [16–18]. Our findings suggest nociplastic pain and fibromyalgia may be highly prevalent among individuals with OUD, and may relate to OUD onset, maintenance, escalation, treatment delay, and pain-precipitated relapse [16–18]. For example, we found that patients with OUD and fibromyalgia had greater odds of strongly agreeing with the statement *‘I am worried pain will cause me to relapse in the future’.* However, limitations of this research were that all participants were recruited from an OUD treatment setting and therefore might not represent the much larger non-treatment-seeking population, and the question inquired about fear of possible future pain-related relapse – rather than asking participants about personal history of prior pain-precipitated relapse. Little is known about pain as a precipitant of prior relapse among non-treatment-seeking people with active OUD. Given that 1) fibromyalgia may be specifically associated with OUD relapse, and 2) many non-treatment-seeking individuals have previously engaged in OUD treatment before experiencing relapse, studies of fibromyalgia and pain-precipitated relapse among non-treatment-seeking individuals are needed.

Therefore, the aim of the present study was to determine if fibromyalgia is associated with increased odds of prior pain-precipitated relapse among non-treatment-seeking individuals with OUD. To achieve this aim, we conducted a cross-sectional survey embedded within a syringe service program that provides comprehensive harm reduction services for people who use substances, including opioids. Specifically, we surveyed individuals with OUD who were not currently seeking or engaged in OUD treatment about their pain and opioid use to assess the sample prevalence of fibromyalgia and determine whether fibromyalgia was associated with increased odds of agreeing with the statement *‘I have tried to stop using opioids before, but pain caused me to relapse’.*

## 2. Methods

### 2.1. Participants

One hundred and fifty-three adult participants were consecutively recruited from Safe Point Equitas Health – a syringe service program offering comprehensive harm reduction services in Columbus, Ohio between January 10, 2023, and April 12, 2023. Eligible participants were those who endorsed opioid use during the past 7 days, met Diagnostic and Statistical Manual 5 (DSM-5) OUD criteria, and reported they were not seeking or engaged in OUD treatment. To avoid incentivizing individuals without pain to falsely report pain to gain participation in the study, potential participants were enrolled irrespective of pain (i.e. pain was not an inclusion criterion). The only exclusion criteria were inability to provide informed consent, read, or understand survey items.

Recruitment was conducted by a trained Research Coordinator while syringe service program clients waited for harm reduction services. Participants accessed survey items on a tablet computer privately and were not allowed to interact during the survey. Survey data were collected using REDCap, a web platform for securely collecting personal health information and managing online databases [23, 24].

No individuals who were offered participation declined. One individual did not meet OUD criteria, five denied opioid use in the last seven days, and five provided no answer regarding opioid use in the last seven days. One participant was missing the primary predictor variable. The final sample was one hundred and forty-one (*n* = 141). The study protocol (reference # 2022H0369) adhered to the Declaration of Helsinki and was approved by the OSUWMC Institutional Review Board on November 27, 2022. Consistent with 45 CFR 46.117(c), a waiver of signed informed consent was granted, as a) the sole record linking the participant to the research would have been their signature on an informed consent form, and b) the primary risk to participants was breach of confidentiality. Therefore, participants provided verbal informed consent and were monetarily compensated for their time.

### 2.2. Measures

#### 2.2.1. Survey

The survey included a validated self-report version of the Diagnostic and Statistical Manual of Mental Disorders, fifth edition (DSM-5) OUD criteria [25–27], demographic information, questions about pain and substance use, and the following validated instruments.

##### 2.2.1.1. Fibromyalgia

Fibromyalgia was assessed by the 2011 American College of Rheumatology Fibromyalgia Survey (ACRFMS). ACRFMS records the location and number of sites of bodily pain (0–19) and the severity of central nervous system-related symptoms including problems thinking, fatigue and difficulty sleeping (0–12). As a continuous scale (range 0–31), ACRFMS has been extensively utilized to determine fibromyalgia severity and as a surrogate measure for nociplastic pain [28–32]. Alternatively, it may be used with a specific cut-off point to indicate the presence of fibromyalgia (ACRFMS ≥ 13, sensitivity 96.6% and specificity 91.8%) [33]. ACRFMS score has previously been shown to be robustly predictive of pain, disability, and treatment outcomes in diverse clinical populations – even when other causes of pain (i.e. rheumatic disease, post-surgical pain, osteoarthritis, etc.) are present [19, 28, 34–39].

##### 2.2.1.2. Mental health (anxiety and depression) and pain severity

Mental health was assessed by the Research and Development (RAND) Corporation RAND 36-Item Health Survey 1.0 (RAND-36) Mental Health domain score [40]. RAND-36 is a widely adopted survey designed to assess health-related quality of life along eight domains: general health, physical functioning, mental health, social functioning, vitality, bodily pain, role limitations due to physical health and role limitations due to emotional problems. Scoring RAND-36 requires linear transformation of each of its 36 items to a range of 0–100 and averaging items by domain [40, 41]. Lower domain scores represent worse health-related quality of life. The validity and reliability of RAND-36 has been studied extensively [41–44]. The RAND-36 Mental Health domain contains 5 questions relating to anxiety and depression, has a reported Cronbach’s α of 0.90, and mean score of 70.38 (SD 21.97) [45]. The Mental Health domain score may detect the probable presence of an anxiety or depressive disorder [46]. A score of 100 indicates optimal mental health. Pain severity was assessed by RAND-36 item 21 which asks *‘How much bodily pain have you had during the past 4 weeks?’* Responses are graded from none to very severe.

##### 2.2.1.3. History of pain-precipitated relapse

An original item was written to assess participants’ perception that pain had caused them to relapse during a prior OUD recovery attempt. We based this item on our previous studies of nociplastic pain and addiction in which we asked treatment-engaged individuals a similar question about fear of pain-precipitated relapse [16–18]. The item was *‘Please indicate whether you strongly agree, agree, feel neutral, disagree, or strongly disagree with the statement: I have tried to stop using opioids before, but pain caused me to relapse’.* Responses were scaled: *strongly disagree (*1*), disagree (*2*), neutral (*3*), agree (*4*) or strongly agree (*5*).*

### 2.3. Analyses

First, descriptive statistics were used to analyze sample demographics, opioid use patterns, OUD severity, mental health, and pain characteristics. Then cumulative odds ordinal logistic regression analyses with proportional odds were conducted to determine whether fibromyalgia and relevant co-variates were associated with increased odds of agreement with the statement *‘I have tried to stop using opioids before, but pain caused me to relapse’* among non-treatment-seeking participants with OUD. **Model 0** was an intercept only model. **Model 1** was a single predictor model with fibromyalgia status as the sole independent variable. **Model 2** was a multiple predictor model with fibromyalgia as the primary predictor and age, sex, RAND-36 Mental Health domain score (depression and anxiety), and OUD severity as covariates. Models 1 and 2 were based on our prior study of fibromyalgia among individuals in OUD treatment [16, 18]. Age, sex, and negative affective states including depression and anxiety have previously been linked to pain and OUD outcomes and were therefore potentially important covariates despite our prior finding that they did not predict fear of pain-precipitated relapse among OUD patients [18, 47–49]. **Model 3** was a multiple predictor model with fibromyalgia and pain severity as predictors. Pain severity was represented by dummy variables for each category (*very mild, mild, moderate, severe, very severe*). Model 3 violated the assumption of proportional odds. Therefore, **Model 4** was run with the same predictor variables as model 3, but with the assumption of proportional odds relaxed for the offending variable (*very severe* pain). **Model 5** was a partial proportional odds ordinal logistic regression similar to Model 4, but with fibromyalgia removed from the list of predictor variables. Model 5 was used to assess how fibromyalgia affected model fit over and above pain severity alone. Regression analyses were conducted in R 4.3.0 with R Studio 2023.06.0 + 421 using *ordinal* and *VGAM* packages [50–53]. All other analyses were completed with IBM SPSS Statistics, version 28.0 [54].

## 3. Results

### 3.1. Sample characteristics

Demographic data were provided by 137 (97.2%) participants. Fifty-eight (42.3%) reported their gender as woman and 75 (54.7%) were men. The mean age was 37.7 (SD = 8.1). One hundred and thirty-three (94.3%) met DSM-5 criteria for severe OUD. Table 1 displays participant characteristics.

**Table 1.** Participant characteristics.

Characteristic	Participants (*n* = 141)/Percent
*Age mean (SD)*	
Years	37.7 (8.1)
*Racial Identity n (%)*	
Black	17 (12.2)
White	114 (82.0)
Any Other Race	4 (2.9)
*Ethnicity n (%)*	
Hispanic	2 (1.5)
Non-Hispanic	123 (89.8)
*Gender n (%)*	
Woman	58 (42.3)
Man	75 (54.7)
Gender diverse	3 (2.2)
Prefer not to say	1 (0.7)
*Bodily pain n (%)*	
No pain (in past 4 weeks)	12 (8.6)
Single-site pain	10 (7.1)
Multi-site pain (2 to 3 sites)	38 (26.9)
Multi-site pain (≥ 4 sites)	64 (45.4)
Fibromyalgia (ACRFMS ≥ 13)	49 (35.0)
*ACRFMS score mean (SD)*	
ACRFMS total	11.3 (6.6)
*OUD severity mean (SD)*	
DSM-5 criteria	9.8 (1.9)
*Age of first opioid use mean (SD)*	
Years	23.7 (9.3)
*Route of opioid administration n (%)*	
Intravenous	117 (84.2)
Intramuscular or subcutaneous	12 (8.5)
Smoking	68 (48.9)
Snorting / sniffing up the nose	37 (26.2)
Swallowing by mouth	12 (8.6)
Another Route	2 (1.4)
*Cost of DAILY OPIOID USE mean (SD)*	
USD ($)	105.5 (113.4)

Note: SD = standard deviation; OUD = opioid use disorder; ACRFMS = American College of Rheumatology Fibromyalgia Survey; USD = United States dollar.

### 3.2. Pain

**Figure 1.**
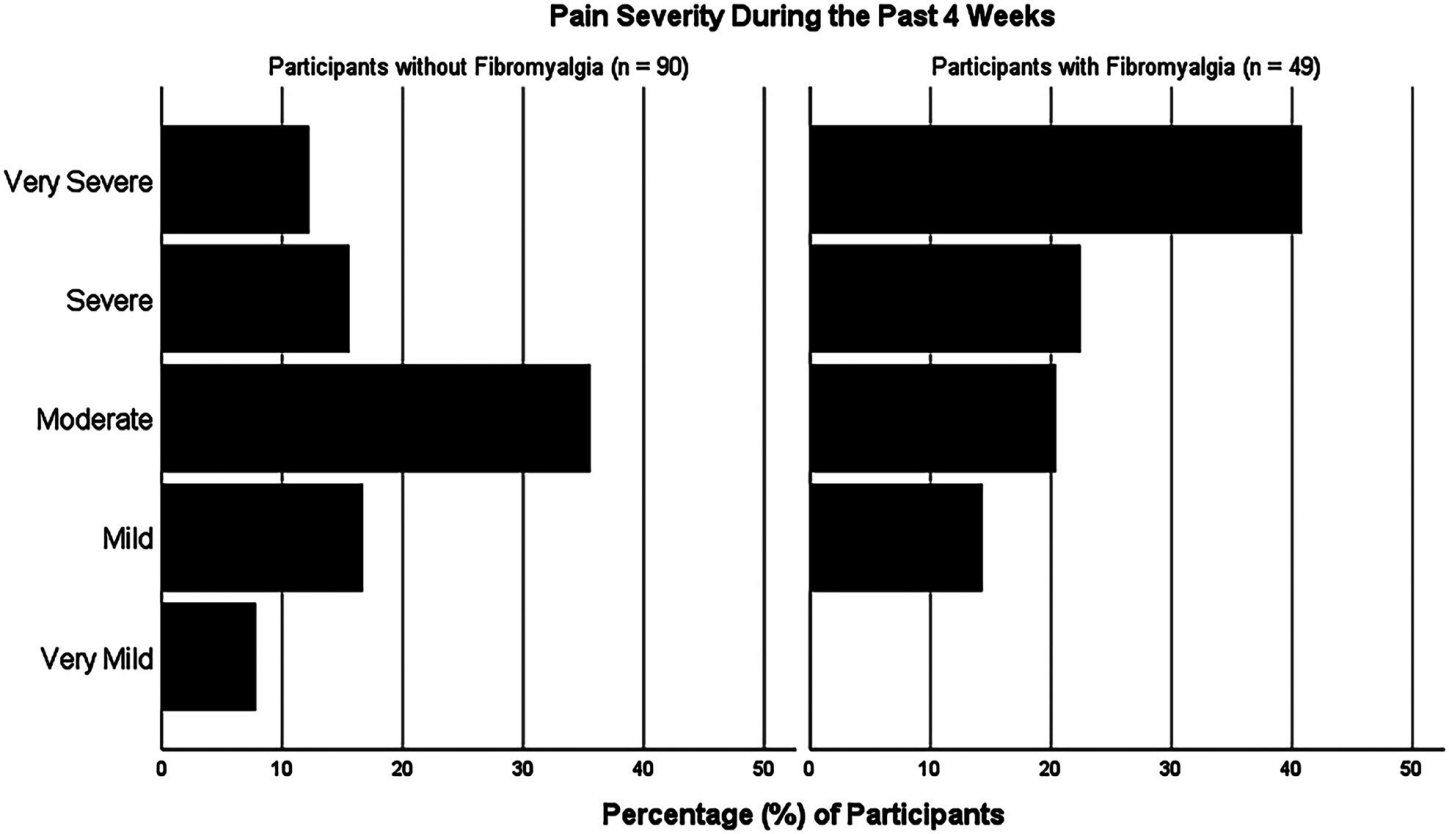
Displays self-reported pain severity over the past 4 weeks.

Bodily pain was notably burdensome in this non-treatment-seeking sample of adults with OUD. Among all participants, 70.5% reported at least moderate pain and the median pain severity over the past 4 weeks was moderate overall with an interquartile range (IQR) of mild to severe on RAND-36. Forty-nine (35.0%) participants met ACRFMS criteria for fibromyalgia. Participants with fibromyalgia did not significantly differ from those without fibromyalgia based on age of first opioid use ([fibromyalgia Mdn = 20.5, No fibromyalgia Mdn = 22.5], *U* = 1866.5, z = −1.314, *p* = 0.189), duration of opioid use ([fibromyalgia Mdn = 14, No fibromyalgia Mdn = 13], *U* = 1788.0, z = −1.295, *p* = 1.95), number of times opioids were used per day ([fibromyalgia Mdn = 5, No fibromyalgia Mdn = 4], *U* = 1893.0, z = −1.581, *p* = 0.114), or amount of money spent on opioids daily ([fibromyalgia Mdn = $90, No fibromyalgia Mdn = $70], *U* = 1904.0, z = −1.246, *p* = 0.213). Median pain severity among participants with fibromyalgia was severe with an IQR of moderate to very severe. Mean total ACRFMS score was 11.3 ± 6.6. The correlation between ACRFMS score and pain severity was (r_s_ (137) = 0.450, *p* < .001), and the nominal-by-nominal association between fibromyalgia and *very severe pain* was (r_phi_ (137) = 0.328, *p* < .001). Figure 1 displays self-reported pain severity over the past 4 weeks.

**Figure 2.**
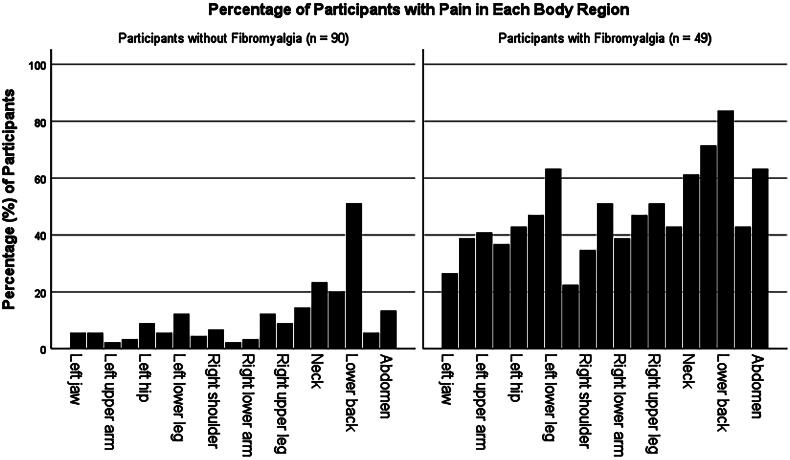
Is a visualization of the distribution of bodily pain among the sample.

Multi-focal pain – a feature of nociplastic pain – was prevalent, with 64 (45.4%) participants reporting pain in four or more body sites. Low back pain was most frequently reported (*n* = 88, 62.4%), followed by upper back pain (*n* = 53, 37.6%) and neck pain (*n* = 51, 36.2%). Non-pain symptoms commonly associated with fibromyalgia and the nociplastic pain phenotype were also common across the sample. These included moderate to severe cognitive problems (thinking or remembering), fatigue, or waking unrefreshed numbered 62 (44.3%), 72 (51.4%) and 86 (61.4%) respectively. One hundred and seven (76.4%) indicated that their pain or other nociplastic pain associated symptoms had lasted 3 months or longer. Eighty-three participants (59.7%) agreed or strongly agreed that pain had previously caused them to relapse. Figure 2 is a visualization of the distribution of bodily pain among the sample.

### 3.3. Regression analyses

Ordinal logistic regression analyses were conducted to determine whether fibromyalgia and relevant co-variates were associated with increased odds of agreement with the *statement ‘I have tried to stop using opioids before, but pain caused me to relapse’.*

#### 3.3.1. Model 1 –single predictor model with fibromyalgia as the sole independent variable

A cumulative odds ordinal logistic regression with proportional odds was run to determine the effect of fibromyalgia on odds of acknowledging prior pain-precipitated OUD relapse. Fibromyalgia was the sole predictor variable included in model 1. The assumption of proportional odds was assessed *via* the nominal test procedure from the ordinal package [50]. An insignificant nominal test showed that the proportional odds assumption was upheld for the predictor fibromyalgia (χ^2^(3) = 3.80, *p* = 0.283). The Brant test also indicated the proportional odds assumption had been met (χ^2^(3) = 5.24, *p* = 0.15). Model 1 statistically significantly predicted the dependent variable over and above the intercept-only model, χ^2^(1) = 13.03, *p* < 0.001. Fibromyalgia was associated with significantly increased odds of agreement with the statement *‘I have tried to stop using opioids before, but pain caused me to relapse’* 3.32 (95% CI, 1.72–6.55).

#### 3.3.2. Model 2 – multiple predictor model with fibromyalgia and covariates age, sex, mental health, and OUD severity

A second cumulative odds ordinal logistic regression with proportional odds was run to determine the effect of fibromyalgia and covariates age, sex, anxiety and depression (RAND-36 Mental Health domain score), and OUD severity (DSM-5 criteria) on odds of agreeing that pain had caused prior OUD relapse. Covariates age and OUD severity were mean centred prior to analysis. The assumption of proportional odds was upheld by an insignificant nominal test result for each of the 5 predictor variables, although age did trend towards significance (χ^2^(3) = 7.54, *p* = 0.06). However, the Brant test also suggested the assumption of proportional odds was met (χ^2^(15) = 22.8, *p* = 0.09). All variance inflation factors were less than 10, confirming the assumption of no multicollinearity was met.

Only fibromyalgia was significantly associated with increased odds of agreement with the statement *‘I have tried to stop using opioids before, but pain caused me to relapse’.* The odds of agreeing with this statement were 2.78 (95% CI, 1.39–5.65) higher among participants with fibromyalgia, a statistically significant effect χ^2^(1) = 2.86, *p* = 0.004. All other independent variables including age 1.03 (95% CI, 0.99–1.07), sex 1.10 (95% CI, 0.59–2.05), anxiety and depression (RAND-36 Mental Health domain score) 0.99 (95% CI, 0.98–1.01) and OUD severity (DSM-5 criteria) 1.12 (95% CI, 0.94–1.34) were insignificant.

Comparison of model fit statistics between model 2 and model 1 suggested that model 1 (fibromyalgia as the sole predictor) was a better fit to the data than model 2 (multiple predictor model with fibromyalgia, age, sex, anxiety, depression, and OUD severity). The model 2 AIC (418.2) was slightly higher than the model 1 AIC (415.7), and a likelihood ratio test comparing the two models showed that the addition of variables age, sex, anxiety, depression, and OUD severity did not improve the fit of model 2 over model 1 (χ^2^(4) = 5.53, *p* = 0.237). Therefore, model 1 (fibromyalgia as the sole predictor) was the most parsimonious model. Table 2 provides a comparison of model statistics.

**Table 2.** Comparison of participants with and without fibromyalgia

Characteristic	Fibromyalgia (*n* = 49)	No Fibromyalgia (*n* = 90)
*Age mean (SD)*	37.9 (8.5)	37.5 (7.9)
*Racial identity n (%)*		
Black	5 (10.2)	12 (13.3)
White	40 (81.6)	74 (82.2)
Any Other Race	1 (2.0)	3 (3.33)
*Ethnicity n (%)*		
Hispanic	1 (2.1)	1 (1.1)
Non-Hispanic	42 (85.7)	81 (91.0)
*Gender n (%)*		
Woman	19 (39.6)	39 (43.8)
Man	26 (54.2)	49 (55.1)
Gender diverse	2 (4.2)	1 (1.1)
Prefer not to say	1 (2.0)	0 (0)
*Route of opioid administration n (%)*		
Intravenous	42 (85.7)	75 (83.3)
Intramuscular or subcutaneous	5 (10.2)	7 (7.8)
Smoking	27 (55.1)	41 (45.6)
Snorting / sniffing up the nose	16 (32.7)	21 (23.3)
Swallowing by mouth	6 (12.2)	6 (6.7)
Another Route	1 (2.0)	1 (1.1)
*ACRFMS total mean (SD)*	18.3 (5.5)	7.5 (3.0)
* Pain Severity median (IQR)	5 (4–6)	4 (3–5)
** Prior Pain-Related Relapse median (IQR)	3 (3–4)	2 (1–3)

Note: SD = standard deviation; IQR = interquartile range; ACRFMS = American College of Rheumatology Fibromyalgia Survey. * Participants were asked *‘How much bodily pain have you had during the past 4 weeks?’* Responses were 1 = none, 2 = very mild, 3 = mild, 4 = moderate, 5 = severe, 6 = very severe. ** Participants were asked *‘Please indicate whether you strongly agree, agree, feel neutral, disagree, or strongly disagree with the statement: I have tried to stop using opioids before, but pain caused me to relapse’.* Responses were 1 = strongly disagree, 2 = disagree, neutral (3), agree (4) or strongly agree (5).

#### 3.3.3. Model 3 – multiple predictor model with fibromyalgia and pain severity

A third cumulative odds ordinal logistic regression with proportional odds was run to determine the effect of fibromyalgia on the odds of agreeing that pain had caused prior OUD relapse after accounting for pain severity. RAND-36 item 21 response categories were converted to dichotomous dummy variables for this analysis. The assumption of proportional odds was violated for the dummy variable representing *very severe pain* per a significant nominal test (χ^2^(3) = 14.2, *p* = 0.003) necessitating the use of an alternative statistical procedure (partial proportional odds model – see model 4).

#### 3.3.4. Model 4 – multiple predictor model with fibromyalgia and pain severity (partial proportional odds)

Because the assumption of proportional odds was violated in model 3, a fourth model with the assumption of proportional odds relaxed for the dummy variable representing *very severe pain* was run. This partial proportional odds ordinal logistic regression included fibromyalgia and pain severity dummy variables as independent variables. Fibromyalgia remained significantly associated with increased odds of agreement with the statement *‘I have tried to stop using opioids before, but pain caused me to relapse’* after accounting for pain severity. The odds of agreeing with this statement were 2.25 (95% CI, 1.10–4.61) higher among participants with fibromyalgia, a statistically significant effect χ^2^(535) = 2.23, *p* = 0.029. *Moderate pain, severe pain*, and *very severe pain* were also associated with acknowledgement of prior pain-precipitated relapse (see Table 2). Because dummy variables for *moderate pain, severe pain*, and *very severe pain* were found to be significant predictors in the partial proportional odds model, a fifth model without fibromyalgia was run to determine whether a better model fit might be achieved with pain severity alone.

#### 3.3.5. Model 5 – multiple predictor model with pain severity only (partial proportional odds)

After removing fibromyalgia from the model, dummy variables for *moderate pain, severe pain*, and *very severe pain* continued to be significantly associated with agreement with *‘I have tried to stop using opioids before, but pain caused me to relapse’.* However, comparison of model fit statistics revealed that removing fibromyalgia adversely impacted model fit. Model 4 (fibromyalgia + pain severity) was a better fit to the data than model 5 (pain severity only). The model 5 AIC (405.6) was higher than the model 4 AIC (402.5), and a likelihood ratio test comparing the two models showed that the removal of fibromyalgia worsened the fit of model 5 relative to model 4 (χ^2^(536) = 5.07, *p* = 0.02). Therefore, model 4 (fibromyalgia + pain severity) was the best fitting model. This result indicated that fibromyalgia was uniquely associated with pain-precipitated relapse even after accounting for pain severity. Table 3 presents the results of each statistical model.

**Table 3.** Model comparison.

Model	Type	AIC	Predictors	OR (CI)	β (CI)
Model 0	PO	426.7	Intercept only	n/a	n/a
Model 1	PO	415.7	* Fibromyalgia	3.32 (1.72–6.55)	1.20 (0.54–1.88)
Model 2	PO	418.2	* Fibromyalgia	2.78 (1.39–5.57)	1.02 (0.33–1.73)
			Age	1.03 (0.99–1.07)	0.03 (−0.01–0.07)
			Sex	1.10 (0.59–2.05)	0.09 (−0.52–0.72)
			Mental Health	0.99 (0.98–1.01)	−0.01 (−0.02–0.01)
			OUD Severity (DSM-5)	1.12 (0.94–1.34)	0.12 (−0.06–0.29)
Model 3	PO	410.7	* Fibromyalgia	2.29 (1.13–4.71)	0.83 (0.12–1.55)
			Very Mild Pain	1.83 (0.36–9.38)	0.60 (−1.02–2.23)
			Mild Pain	1.39 (0.38–5.01)	0.33 (−1.00–1.61)
			Moderate Pain	2.96 (0.92–9.55)	1.06 (−0.08–2.26)
			*Severe Pain	6.14 (1.65–23.4)	1.82 (0.50–3.15)
			*Very Severe Pain	6.79 (1.75–26.7)	1.92 (0.56–3.29)
Model 4	PPO	402.5	* Fibromyalgia	2.25 (1.10–4.61)	0.81 (0.10–1.53)
			Very Mild Pain	2.10 (0.40–11.2)	0.74 (−0.93–2.41)
			Mild Pain	1.61 (0.45–5.78)	0.48 (−0.80–1.75)
			* Moderate Pain	3.71 (1.14–12.1)	1.31 (0.13–2.49)
			* Severe Pain	8.32 (2.20–31.6)	2.11 (0.79–3.45)
			Very Severe Pain (P[Y ≥ 2])	0.89 (0.19–4.04)	−0.12 (−1.64–1.40)
			Very Severe Pain (P[Y ≥ 3])	3.41 (0.83–14.1)	1.22 (−0.19–2.65)
			* Very Severe Pain (P[Y ≥ 4])	6.77 (1.66–27.5)	1.91 (0.51–3.31)
			* Very Severe Pain (P[Y ≥ 5])	13.0 (3.21–52.2)	2.56 (1.17–3.96)
Model 5 (Pain severity only)	PPO	405.6	Very Mild Pain	2.01 (0.38–10.7)	0.70 (−0.97–2.37)
			Mild Pain	2.07 (0.58–7.35)	0.73 (−0.54–2.00)
			*Moderate Pain	4.28 (1.32–13.9)	1.5 (0.27–2.63)
			*Severe Pain	11.5 (3.06–42.8)	2.44 (1.12–3.76)
			Very Severe Pain (P[Y ≥ 2])	1.36 (0.31–5.89)	0.31 (−1.16–1.77)
			*Very Severe Pain (P[Y ≥ 3])	5.23 (1.33–20.6)	1.65 (0.28–3.02)
			*Very Severe Pain (P[Y ≥ 4])	10.2 (2.62–39.8)	2.32 (0.96–3.68)
			*Very Severe Pain (P[Y ≥ 5])	19.5 (5.03–75.5)	2.97 (1.62–4.32)

*Notes:* AIC = Akaike Information Criterion, lower AIC indicates better model fit; OR = Odds Ratio reflecting the odds of agreement with the statement *‘I have tried to stop using opioids before, but pain caused me to relapse’*; CI = 95% Confidence Interval; PO = Proportional Odds Ordinal Logistic Regression; PPO = Partial Proportional Odds Ordinal Logistic Regression; P[Y ≥] = Because the predictor Very Severe Pain is allowed to vary across dependent variable response categories in the PPO model, and because the dependent variable has 5 possible responses, 4 sub-models are fitted as represented in the table. Pain Severity categories are dummy coded responses to the Research and Development Corporation 36-Item Health Survey (RAND-36) item 21 which asks respondents *‘How much bodily pain have you had during the past 4 weeks?’* * = Statistically significant predictor in model (*p* < 0.05).

**Figure 3.**
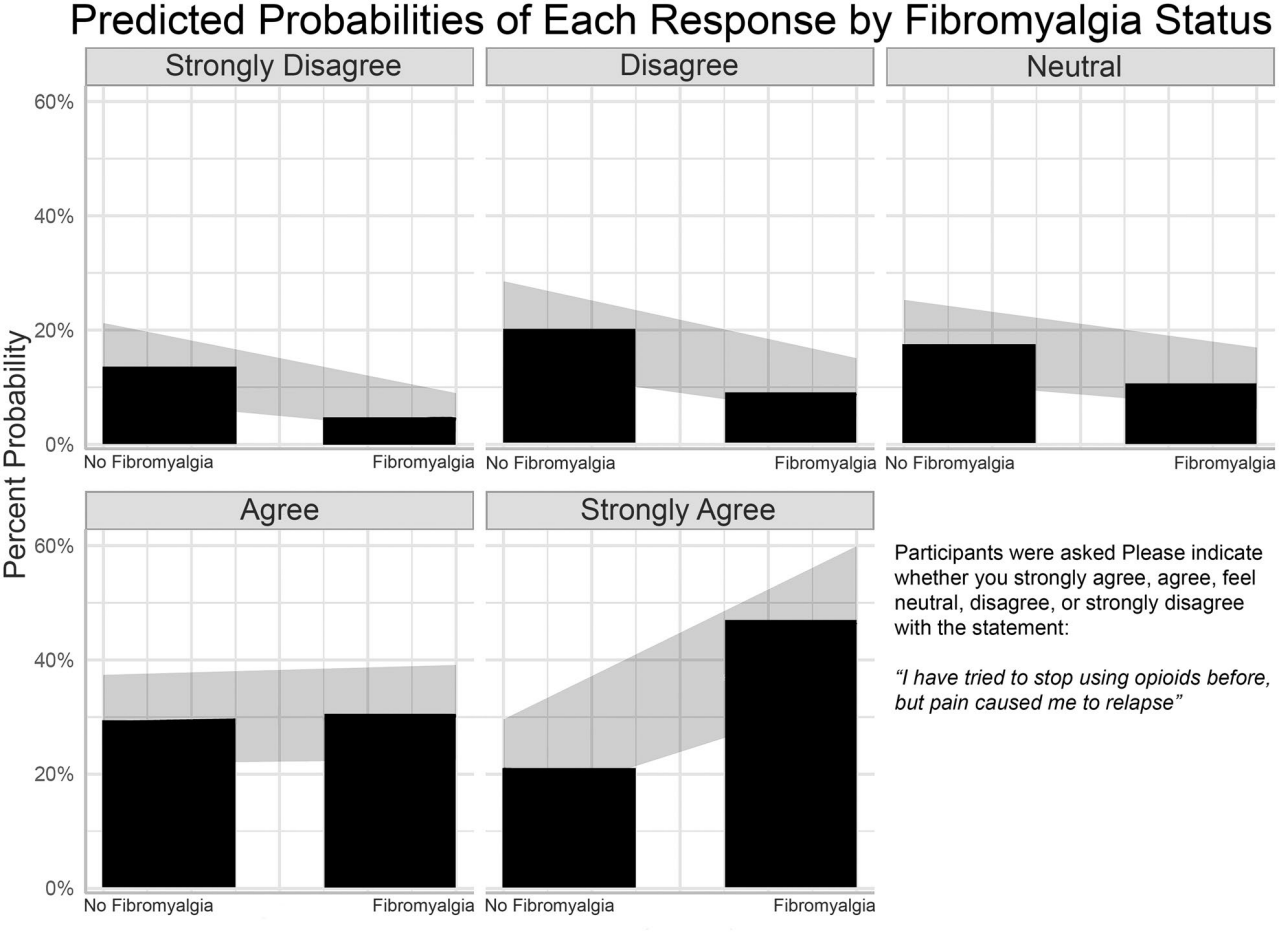
Displays the predicted probabilities of each possible response to the question ‘*please indicate whether you strongly agree, agree, feel neutral, disagree, or strongly disagree with the statement: I have tried to stop using opioids before, but pain caused me to relapse’* among participants with and without fibromyalgia.

Figure 3 displays the predicted probabilities of each possible response to the question *‘Please indicate whether you strongly agree, agree, feel neutral, disagree, or strongly disagree with the statement: I have tried to stop using opioids before, but pain caused me to relapse’* among participants with and without fibromyalgia.

## 4. Discussion

Thirty-five percent of our sample met criteria for fibromyalgia and meeting criteria was associated with 232% greater odds of strongly agreeing with the statement ‘*I have tried to stop using opioids before, but pain caused me to relapse’.* This is the first study to demonstrate a relationship between fibromyalgia and history of pain-precipitated relapse among non-treatment-seeking individuals with OUD. Given that over 80% of those living with OUD in 2022 received no MOUD treatment, and that many non-treatment-seeking individuals – including 60% of participants in this study – have previously experienced pain-precipitated relapse, fibromyalgia may be an important unrecognized factor that could impede OUD treatment engagement [1].

The addition of pain severity into models 3 and 4 led to a reduction in the strength of the effect of fibromyalgia on pain-precipitated relapse. However, this should not be interpreted as diminishing the importance of fibromyalgia on this outcome because of the logical relationship between fibromyalgia and pain severity. Severe pain is neither necessary nor sufficient to cause fibromyalgia, but fibromyalgia is sufficient to cause severe pain. We found that the total fibromyalgia survey criteria (ACRFMS) score was positively associated with pain severity (r_s_ (137) = 0.450, *p* < .001), and the nominal-by-nominal association between having fibromyalgia and reporting very severe pain was (r_phi_ (137) = 0.328, *p* < .001). Therefore, fibromyalgia very likely accounted for a substantial proportion of variance in pain-precipitated relapse attributed to pain severity in models 3 and 4. The present results do not preclude important relationships between other pain conditions and pain-precipitated relapse. However, congruent with prior evidence of neurobiological overlap between fibromyalgia and the brain disease model of addiction, this report provides new evidence that fibromyalgia may be specifically associated with OUD relapse.

The results of the present study align with prior pain and addiction research. It has been shown that individuals with nociplastic pain conditions such as fibromyalgia may be less responsive to the analgesic effects of opioids yet are more likely to engage in chronic opioid use following surgery [34, 38, 55–57]. Additionally, studies have suggested that individuals with nociplastic pain may over-produce endorphins, leading to a state of endogenous opioid induced hyperalgesia [58, 59]. By extension, the presence of nociplastic pain may neurobiologically alter OUD in a plethora of ways, not the least of which might be altering responsiveness to MOUD treatment. Therefore, it is plausible that the underlying neurobiology of nociplastic pain may represent a vulnerability to pain-precipitated relapse of OUD.

Our group previously found that nociplastic pain and fibromyalgia were associated with self-report of pain as reason for OUD onset, maintenance, escalation, OUD treatment delay, and OUD relapse among treatment-engaged individuals with OUD [16, 18]. In one such study, fibromyalgia was associated with 151% greater odds of agreement with the statement *‘I am worried pain will cause me to relapse in the future’,* while theoretically related covariates age, sex, anxiety, depression, and OUD severity had no significant effect on the odds of fearing pain-precipitated relapse [18]. The present study builds upon this foundation in two important ways. First, rather than asking participants to forecast the risk of a hypothetical future event, the present study was more concrete in that it inquired about prior experience of pain-precipitated relapse. Second, our prior studies recruited treatment-engaged participants from an OUD treatment centre, whereas the present research recruited non-treatment-seeking individuals from a syringe service program. We have replicated and extended our prior findings to the majority of people living with OUD who are non-treatment-seeking.

Prior research has identified neurobiological overlap between CNS mechanisms implicated in the brain disease model of addiction and nociplastic pain conditions such as fibromyalgia [60–62]. The brain disease model of addiction involves progressively worsening dysfunction of the mesolimbic dopamine system. Nociplastic pain may also be associated with dysfunction of the mesolimbic dopamine system including lower D2 receptor binding and dopamine activity in the dorsal striatum [63], and reward processing is altered in both conditions [64]. Nociplastic pain and opioid use are also associated with perturbations of the endogenous opioid system affecting pain perception and reward sensitivity [65]. Similarities in endogenous opioid dysfunction in nociplastic pain and OUD may have a neurogenetic basis, as polymorphisms of the OPRM1 gene are associated with both nociplastic pain and OUD [66–70]. Dysregulation of the central nucleus of the amygdala (CeA), which has been referred to as the ‘nociceptive amygdala’ for its role in the emotional processing of pain plays a key role in the brain disease model of addiction [71, 72]. CeA neuroadaptation in the brain disease model of addiction includes up-regulation of pro-nociceptive neurotransmitter corticotropin-releasing factor (CRF). Increased cerebrospinal fluid CRF concentration has been associated with pain severity in subjects with nociplastic pain [73]. Additionally, persistent activation of the amygdala by chronic pain has been observed to alter medial prefrontal cortex (mPFC) functioning, causing deficits including in executive functioning [74–78]. Cognitive deficits, one of the non-pain symptoms associated with nociplastic pain and fibromyalgia (aka ‘fibro fog’) may further impair decision making and increase the risk of opioid misuse [79–81]. Taken together, these studies support our contention that nociplastic pain and fibromyalgia, more specifically, may have special relevance for OUD relapse. Clinical and community-based studies, such as the present report, represent a necessary step towards translation of basic neuroscience in this area.

The current research had strengths including low levels of missing data, a relatively large sample size given the inherent difficulty of surveying non-treatment-seeking individuals with OUD, and use of a validated self-report instrument assessing fibromyalgia. However, there were also notable limitations. First, a cross-sectional design precluded the evaluation of temporal relationships (causation), as well as the assessment of long-term outcomes related to pain and opioid use. More robust assessment of treatment history and longitudinal outcomes is essential. Therefore, these preliminary findings require replication in future research. Second, self-report research is subject to recall bias. Our use of specific language and structuring of response categories as a continuum of responses from ‘strongly disagree’ to ‘strongly agree’ rather than a binary ‘yes’ or ‘no’ were intended to mitigate this risk. Third, given recruitment took place at a single syringe service program in a large Midwestern city, external validity is unclear. Our sample consisted largely of non-Hispanic Whites; however, alignment of the current findings with our prior pain and addiction research is reassuring [16, 18].

The present study has clinical and research implications. The US remains in the midst of a grave and sustained opioid overdose epidemic [82]. MOUDs methadone and buprenorphine are effective at increasing treatment retention and reducing the risk of death from accidental opioid overdose [83, 84]. Nevertheless, rates of OUD relapse are high, particularly among patients with co-morbid chronic pain [10, 15, 85]. Additionally, a substantial proportion of non-treatment-seeking individuals report prior episodes of MOUD treatment ending in relapse [8, 9]. The optimal duration for MOUD treatment has yet to be established, but treatment less than 90 days is of limited effectiveness and longer retention is associated with better outcomes [86]. Alternatively, relapse and discontinuation of MOUD are associated with accidental overdose [87]. Together, these facts underscore the importance of finding specific, treatable factors associated with OUD relapse and poor treatment engagement. Our findings suggest that fibromyalgia may be one such factor.

If fibromyalgia is associated with increased odds of OUD relapse, assessment and treatment of fibromyalgia might improve OUD treatment retention. In this study, 35% of non-treatment-seeking individuals with OUD met criteria for fibromyalgia and fibromyalgia was associated with significantly increased odds of endorsing history of pain-precipitated relapse. Our recent related study found that 31.2% of OUD patients met criteria for fibromyalgia and fibromyalgia was associated with fear of pain causing a future relapse [16, 18]. Longitudinal research is needed to confirm whether fibromyalgia is a risk factor for OUD relapse. It is possible that evidence-based treatment of fibromyalgia might enhance OUD treatment engagement and outcomes, although additional research is needed.

The present study found that among non-treatment-seeking individuals with OUD, fibromyalgia was associated with substantially greater odds of strong agreement with the statement ‘*I have tried to stop using opioids before, but pain caused me to relapse’.* Specifically, participants with fibromyalgia and OUD had 232% greater odds of strongly agreeing pain had previously caused them to relapse. This finding aligns with a growing body of literature implicating fibromyalgia, and the larger pain phenotype, nociplastic pain, to which fibromyalgia belongs, as complicating factors in OUD.

## Data Availability

Data and R script are available to the Editors upon request. We intend to publish additional papers from this data set and wish to wait until these manuscripts are published before making the dataset fully public.

## Abbreviations

OUD: opioid use disorder
MOUD: medication for opioid use disorder
ACRFMS: American College of Rheumatology 2011 Fibromyalgia Survey Criteria
DSM-5: Diagnostic and Statistical Manual of Mental Disorders, Fifth Edition
RAND-36: RAND 36-item health survey 1.0
CeA: central nucleus of the amygdala
CNS: central nervous system
mPFC: medial prefrontal cortex
CRF: corticotropin-releasing factor
